# Perinatal Lead Exposure Promotes Sex-Specific Epigenetic Programming of Disease-Relevant Pathways in Mouse Heart

**DOI:** 10.3390/toxics11010085

**Published:** 2023-01-16

**Authors:** Laurie K. Svoboda, Kai Wang, Jaclyn M. Goodrich, Tamara R. Jones, Justin A. Colacino, Karen E. Peterson, Martha M. Tellez-Rojo, Maureen A. Sartor, Dana C. Dolinoy

**Affiliations:** 1 Environmental Health Sciences, University of Michigan School of Public Health, Ann Arbor, MI 48109, USA; 2 Department of Computational Medicine and Bioinformatics, University of Michigan Medical School, Ann Arbor, MI 48109, USA; 3 Nutritional Sciences, University of Michigan School of Public Health, Ann Arbor, MI 48109, USA; 4 Center for Research on Nutrition and Health, National Institute of Public Health, Cuernavaca 62100, Mexico; 5 Department of Biostatistics, University of Michigan School of Public Health, Ann Arbor, MI 48109, USA

**Keywords:** heart, cardiovascular disease, DNA methylation, epigenetic, Developmental Origins of Health and Disease (DOHaD), toxicoepigenetics, sex differences

## Abstract

Environmental contaminants such as the metal lead (Pb) are associated with cardiovascular disease, but the underlying molecular mechanisms are poorly understood. In particular, little is known about how exposure to Pb during early development impacts the cardiac epigenome at any point across the life course and potential differences between sexes. In a mouse model of human-relevant perinatal exposures, we utilized RNA-seq and Enhanced Reduced Representation Bisulfite Sequencing (ERRBS) to investigate the effects of Pb exposure during gestation and lactation on gene expression and DNA methylation, respectively, in the hearts of male and female mice at weaning. For ERRBS, we identified differentially methylated CpGs (DMCs) or differentially methylated 1000 bp regions (DMRs) based on a minimum absolute change in methylation of 10% and an FDR < 0.05. For gene expression data, an FDR < 0.05 was considered significant. No individual genes met the FDR cutoff for gene expression; however, we found that Pb exposure leads to significant changes in the expression of gene pathways relevant to cardiovascular development and disease. We further found that Pb promotes sex-specific changes in DNA methylation at hundreds of gene loci (280 DMCs and 99 DMRs in males, 189 DMCs and 121 DMRs in females), and pathway analysis revealed that these CpGs and regions collectively function in embryonic development. In males, differential methylation also occurred at genes related to immune function and metabolism. We then investigated whether genes exhibiting differential methylation at weaning were also differentially methylated in hearts from a cohort of Pb-exposed mice at adulthood. We found that a single gene, Galnt2, showed differential methylation in both sexes and time points. In a human cohort investigating the influence of prenatal Pb exposure on the epigenome, we also observed an inverse association between first trimester Pb concentrations and adolescent blood leukocyte DNA methylation at a locus in GALNT2, suggesting that this gene may represent a biomarker of Pb exposure across species. Together, these data, across two time points in mice and in a human birth cohort study, collectively demonstrate that Pb exposure promotes sex-specific programming of the cardiac epigenome, and provide potential mechanistic insight into how Pb causes cardiovascular disease.

## 1. Introduction

Despite significant advances in therapy and management, cardiovascular diseases (CVDs, e.g., myocardial infarction, coronary artery disease, cardiomyopathy, cardiac arrhythmia, and stroke) remain a leading cause of morbidity and death in the developed world. Moreover, accumulating evidence supports a critical role for environmental exposures in CVD risk and severity [1]. To date, the strongest evidence for environmental factors in the etiology of CVD is available for air pollution, particulate matter, tobacco smoke, and metals (lead, cadmium, arsenic and copper) [1]. The Developmental Origins of Health and Disease hypothesis posits that environmental exposures during critical windows of vulnerability affect the long-term risk of disease [2]. Normal development is highly sensitive to environmental and nutritional factors, and insults during this critical window of vulnerability can have long-term health consequences. Indeed, many studies link an adverse maternal environment to poor cardiovascular outcomes [3,4]. Several mechanisms by which developmental environment can influence long-term cardiovascular health have been identified, including alterations to placental structure and function, hemodynamic changes, disruption in cardiovascular structure and cell number, and disruptions in hormonal signaling, among others [4]. One important molecular mechanism underlying the adverse effects of developmental exposures is altered epigenetic programming. The term epigenetics encompasses heritable factors regulating gene expression without changes to the DNA sequence itself [5]. Normal development and tissue differentiation are characterized by precisely orchestrated spatio-temporal changes in gene expression, and these processes are regulated in part by epigenetic mechanisms [5]. Epigenetic factors include histone modifications, non-coding RNA regulatory mechanisms, and DNA methylation [5,6,7]. DNA methylation, or the addition of a methyl group to the 5- position on cytosine bases, is one of the most widely studied and well-characterized epigenetic factors [8]. CVDs are accompanied by altered DNA methylation, and DNA methylation at various loci is associated with CVD risk [9,10,11,12,13,14].

Pb is a toxic metal that occurs naturally in the earth’s crust, and widespread industrial and commercial use worldwide has resulted in substantial contamination of the environment, including plants, animals, and humans [15]. Common sources of environmental Pb contamination include smelting, mining and recycling of e-waste [16]. Legacy contamination from Pb-based paints and gasoline is also a problem worldwide [16]. Common sources of Pb exposure in the US include drinking water, household dust from Pb-based paint, imported consumer products, and industrial exposures [16]. In 2021, the CDC reference value for blood Pb was lowered from 5 μg/dL to 3.5 μg/dL. This value reflects the 97.5th percentile for blood lead levels in children ages 1–5 and is based on data from the National Health and Nutrition Examination Survey (NHANES) 2015–2016 and 2017–2018 cycles. Although blood lead levels have declined over time, they remain high in some parts of the US and around the world. Recent evidence suggests that the contribution of Pb to cardiovascular disease is greater than previously recognized [17]. Pb exposure in adults is linked to high blood pressure, coronary artery disease, left ventricular hypertrophy, heart failure, and cardiac arrhythmias in humans and animals [17,18,19,20,21,22]. The effects of Pb exposure during the perinatal period and childhood on cardiac health and disease have not been as extensively studied, although several studies demonstrate adverse effects. In animal models, developmental Pb exposure promotes high blood pressure, altered heart rate, increased sensitivity to norepinephrine-induced arrhythmia in adulthood, as well as exacerbation of the normal degenerative effects of aging on the heart [23,24,25,26,27]. In humans, early-life Pb exposure is associated with increased risk of congenital heart defects, impaired left ventricle structure and function, and high blood pressure [28,29,30]. Importantly, these adverse effects in humans were measured in childhood, and the effects of developmental Pb exposure on cardiovascular health in adulthood and beyond have not been investigated. Mechanistically, studies in vitro and in vivo suggest that the cardiovascular effects of Pb are attributed to endothelial dysfunction, modulation of autonomic nervous system activity, altered ion channel function, oxidative stress, and inflammation [19,22,24,31]. 

CVDs exhibit marked sexual dimorphism in their incidence, presentation, and underlying biology [32]. Indeed, conditions such as ischemic heart disease, heart failure, cardiac arrhythmias, and cardiomyopathies are experienced by both sexes but with differences in prevalence, pathogenesis, and prognosis [32]. Notably, females are more susceptible to drug-induced arrhythmias [33], highlighting potential sex specificity in the effects of exogenous agents on cardiovascular health. In spite of this, the sex-specific effects of environmental toxicants such as Pb on cardiovascular health are poorly understood. Developmental Pb exposure induces sex-specific changes in DNA methylation in other tissues [34,35]. However, the sex-specific effects of developmental Pb exposure on the cardiac epigenome require further investigation. We previously reported that Pb exposure during gestation and lactation leads to sex-specific changes in DNA methylation in offspring mouse hearts at 5 months of age. Given that CVD risk can be programmed early in development and manifest as overt disease in adulthood [4,36,37], we hypothesized that we would observe changes in DNA methylation at a subset of disease-related genes in both early life (weaning) and adulthood (5 months of age). To this end, in this work, we examine how Pb exposure during gestation and lactation affects DNA methylation and gene expression in male and female mouse hearts at weaning. We further investigate whether changes in DNA methylation at weaning and adulthood occur at similar loci and whether Pb-affected gene pathways are related to CVD risk and progression. Finally, we explore whether a potential biomarker of Pb exposure, persisting across time in the mice from weaning to early adulthood, is associated with prenatal Pb in human adolescent blood leukocyte samples.

## 2. Materials and Methods

### 2.1. Animals and Study Paradigm

**Figure 1:**
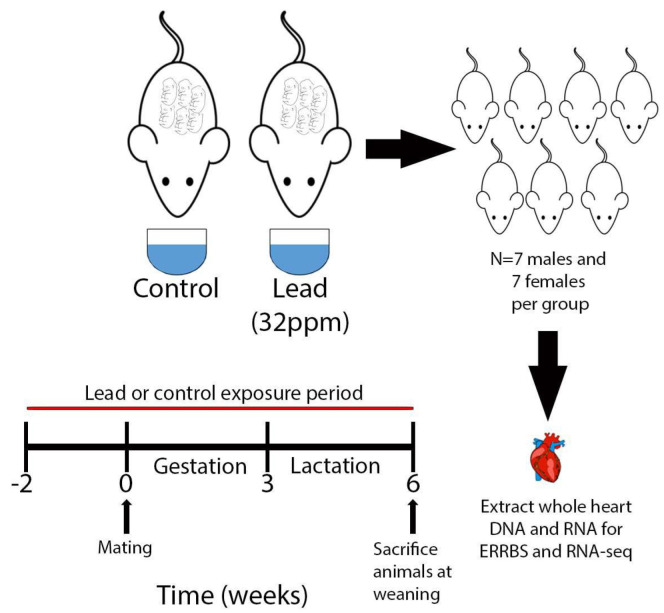
Treatment paradigm for developmental exposure to Pb. Dam exposure began 2 weeks prior to mating and continued through gestation and lactation. Animals were sacrificed at weaning on postnatal day 21. Pb exposure occurred via drinking water, which was administered ad libitum. Whole hearts were harvested and snap-frozen in liquid nitrogen prior to extraction of DNA and RNA.

This work was a part of a much larger study conducted by the National Institute of Environmental Health Sciences (NIEHS) Toxicant Exposures and Responses by Genomic and Epigenomic Regulators of Transcription (TaRGET II) Consortium [38,39]. Mice utilized in this study, as well as the protocol for Pb exposure, were described previously [39]. Briefly, virgin a/a females from a genetically invariant background with 93% identity to C57BL/6 J (6–8 wks old) were mated with virgin a/a males (7–9 wks old), and randomly assigned to receive control or Pb in their drinking water. A concentration of 32 ppm of Pb-acetate was utilized to model human-relevant maternal exposure in the range of 16–60 µg/dL blood Pb levels [35,39]. This range of doses reflects blood Pb levels frequently found in children and adults born in the US during the 1960s and 1970s, as well as among Pb-contaminated communities currently [40,41,42,43]. Pb-acetate was dissolved in distilled water and the concentration was verified using inductively coupled plasma mass spectrometry with a limit of detection of 1.0 µg/L (ICPMS; NSF International, Ann Arbor, MI, USA). Dams were exposed to either Pb-contaminated drinking water or control water for two weeks prior to mating, continuing through gestation and lactation. All animals were fed a phytoestrogen-free modified AIN-93G diet (TD.95092, 7% Corn Oil Diet, Envigo, Indianapolis, IN, USA) for the entire study. At weaning on postnatal day 21, approximately 1 male and 1 female offspring per litter (n = 7 mice per condition) were sacrificed for tissue harvest. A second cohort of mice (approximately 1 male and 1 female per litter) was sacrificed at 5 months of age (n = 6 mice per condition), and data from these mice have been published previously [44]. All animals had access to food and drinking water ad libitum throughout the experiment while housed in polycarbonate-free cages. The treatment paradigm is illustrated in Figure 1. The study protocol was approved by the University of Michigan Institutional Animal Care and Use Committee (IACUC) protocol # PRO00009800.

### 2.2. Tissue Collection and Extraction of RNA and DNA

Body weights were measured for each mouse at sacrifice (Mettler Toledo, Columbus, OH, USA). Upon euthanasia, heart samples were collected according to protocols established by the TaRGET II Consortium (Figure 1 and Ref. [39]). Briefly, after a 6-h fast, euthanasia was carried out via CO_2_ asphyxiation and bilateral pneumothorax. Blood was removed by cardiac puncture, followed by whole-body perfusion with cell culture grade 0.9% saline solution (Sigma Life Sciences, St. Louis, MO, USA). Hearts were immediately snap-frozen in liquid nitrogen and stored at −80 °C. Prior to DNA and RNA extraction, whole hearts were cryo-pulverized to ensure homogeneity across samples. DNA and RNA were extracted using the AllPrep DNA/RNA/Protein mini kit (Qiagen #80004, Hilden, Germany).

### 2.3. Gene Expression Analysis

RNA-seq library preparation and sequencing were performed at the University of Michigan Advanced Genomics Core (N = 7 animals per sex, per condition). For library preparation, we utilized the KAPA mRNA Hyper Prep Kit with Dual Indexing Adapters (Roche, Indianapolis, IN, USA) following manufacturer instructions. We utilized the Agilent 2200 TapeStation to confirm the quantity and quality of the prepared libraries. Sequencing was conducted on the Illumina NovaSeq 6000 in the S2 flow cell, generating paired-end 50 base pair reads. We performed trimming and assessed the quality of sequenced reads using Trim Galore [45] and FastQC [46], respectively, with default parameters. Reads were aligned using STAR, with default parameters [47]. We obtained normalized read counts for each gene, Pb-exposed vs. control, using the TMM method of edgeR [48]. Analyses were stratified by sex. Pathway analysis was conducted for each sex individually utilizing the RNA-enrich method [49], with a directional test and an FDR <0.05 considered statistically significant.

### 2.4. Enhanced Reduced Representation Bisulfite Sequencing

Enhanced Reduced Representation Bisulfite Sequencing (ERRBS) was utilized to examine changes in DNA methylation with Pb exposure in this study (N = 7 per sex, per condition). Our previously published study of animals at 5 months of age included 6 animals per sex, per condition [44]. ERRBS permits the quantitative detection of base-pair resolution DNA methylation at CpG-rich regions in the genome [50]. This technique was performed at the University of Michigan Epigenomics and Advanced Genomics Cores exactly as described previously [39,50,51], and covered 5% of all CpGs in the mouse genome. 50 ng of genomic DNA was utilized for each sample, and DNA quality was assessed using the Qubit (ThermoFisher, Waltham, MA, USA) and 2200 TapeStation systems (Agilent Technologies, Santa Clara, CA, USA), respectively. All samples met the quality standard for next-generation sequencing library preparation. Bisulfite conversion efficiencies for all samples were greater than 99%. Sequencing was conducted on the Illumina NovaSeq 6000 using an S1 100-cycle flowcell. The total alignment percentages ranged from 62.7–68.6%.

### 2.5. Bioinformatics Analysis of ERRBS Data

Quality control, trimming, alignment and methylation calling were conducted exactly as outlined previously [44]. Briefly, FastQC (v0.11.3), TrimGalore (v0.4.5), and Bismark 25 (v0.19.0) were utilized for these steps. Trimming was done for bases with a quality score lower than 20, adapter sequences (required overlap of 6 bp), and end-repair bases from the 3’ end of reads. For alignment and methylation calling, Bowtie2 (v2.3.4) was used to align reads to the mouse mm10 genome. A read depth of at least 5 was required for methylation calls. Differentially methylated CpGs and/or regions were identified using the R Bioconductor package methylSig (v0.5.0). The methylSigDSS() function was utilized to determine differential methylation for Pb vs. control [52], with run included in the model as a covariate to control for batch effects. CpGs with read coverage >1000 (likely the result of PCR amplification) or <10 (decreased power for differential methylation analysis) were removed. The methylSigDSS function was performed across individual CpGs, as well as 1000 base pair regions. We considered an absolute change in DNA methylation of at least 10% and a FDR < 0.05 to be significant. Differentially methylated cytosines (DMCs) or regions (DMRs) were annotated using the R Bioconductor package annotatr (v1.5.9) as outlined previously [53]. We utilized destranding to combine opposite strand CpGs at the same position. For differential methylation testing on individual CpG sites (DMCs), we required a minimum of 4 samples from the control group and 4 samples from the Pb group to have sufficient sequencing coverage.

### 2.6. Gene Set Enrichment Analysis of RNA-seq and ERRBS Data

Gene Ontology, including Biological Process, Cellular Component, and Molecular Function, were used for Gene Set Enrichment Analysis (GSEA). For RNA-seq results, we used the RNA-Enrich Option of LR-Path (http://lrpath.ncibi.org/), with default parameters and a directional test [49,54], to find significantly enriched GO terms. Male and female results were analyzed individually. For ERRBS results, we focused on DMCs, again analyzing males and females separately. To assess the biological pathways enriched among the DMCs, we used Poly-Enrich [55] with the following parameters: locusdef = 10 kb, min_geneset_size = 15, max_geneset_size = 2000. All DMCs with a *p*-value of <0.001 were included in the analysis. Enriched pathways with FDR <0.05 were considered statistically significant.

### 2.7. Analysis of Overlap in DNA Methylation between Sexes

To assess overlap in DNA methylation changes between sexes, we first compared the specific chromosomal locations showing differential methylation with Pb exposure in both males and females but found no directly overlapping sites. We then compared annotated lists of DMCs and DMRs between sexes to identify a list of genes in common between sexes. The hypergeometric test was used to determine the statistical significance of overlap between lists of genes.

### 2.8. Validating a Potential Pb-Biomarker in a Human Study

One gene, *Galnt2*, showed promise as a persistent biomarker of early life Pb exposure in both sexes in mice, at weaning and in early adulthood. To assess the potential human relevance of this finding, we examined whether prenatal Pb exposure was associated with DNA methylation of this gene in human adolescents, a life stage falling within the age range evaluated in the mice. Given the importance of early pregnancy exposure on widespread epigenetic programming [56], we investigated associations with first-trimester maternal blood Pb levels. Briefly, participants were part of the second and third birth cohorts of the longitudinal study, Early Life Exposures in Mexico to Environmental Toxicants (ELEMENT). Full cohort details have been previously detailed, including information on participants with epigenetic data in adolescence [57,58,59]. Out of 526 participants followed-up from birth to adolescence, 365 had blood Pb concentrations measured in samples collected from their mothers during the first trimester of pregnancy. Among these participants, we examined the association between first-trimester Pb with DNA methylation at 146 CpG sites annotated to *GALNT2* (chr1:230,193,536-230,417,875; genome build GRCh37/hg19). Data were extracted from Infinium MethylationEPIC array data [60] generated using blood leukocyte DNA collected during adolescence (participants ages 11–18 years at time of sample collection). We previously described quality control and data normalization procedures [58]. Linear regression models with DNA methylation at each *GALNT2* CpG site as the outcome and first-trimester maternal blood Pb concentration (natural-log transformed) as the predictor were run, adjusting for covariates. Covariates included gender, batch, and estimated cell-type proportions (granulocytes, monocytes, B-cells, and CD4+ T-cells). We also ran a sensitivity analysis adjusting for original cohort and maternal smoking during pregnancy. Results were considered statistically significant at a Bonferroni corrected *p*-value (<0.0003).

## 3. Results

**Figure 2:**
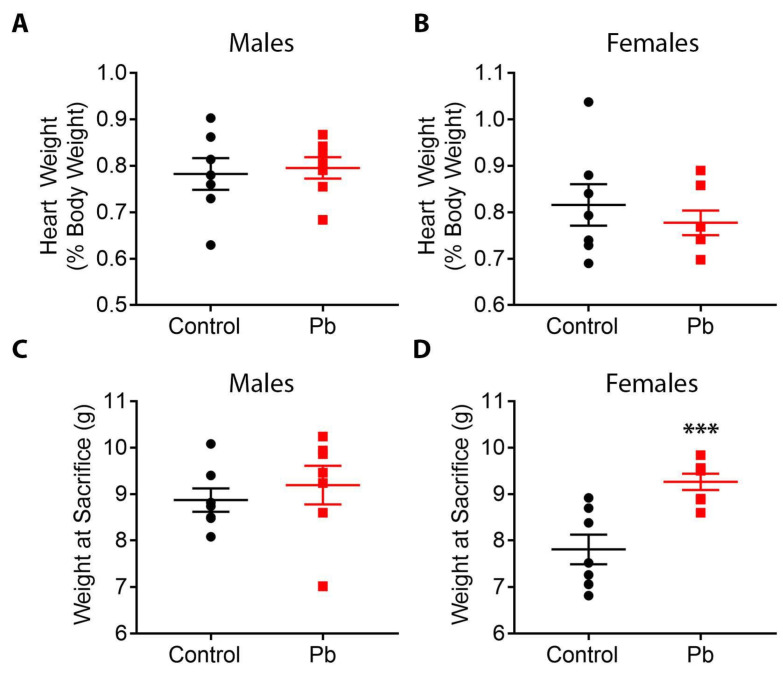
Heart weights as a percentage of body weight (**A**,**B**) and total body weights (**C**,**D**) for male and female offspring at sacrifice. N = 7 animals per group. *** *p* < 0.001. Linear mixed effects regression, with litter-specific random effects to account for within-litter correlation, was used to determine statistical significance.

Perinatal exposure to Pb had no significant effect on litter size, pup mortality, or the percentage of males vs. females [39]. Heart weights as a percentage of total body weight did not differ between Pb and control animals for either males or females; however, Pb-treated females were significantly heavier at weaning compared to control females. No significant weight changes were observed in males (Figure 2).

### 3.1. Effect of Perinatal Pb Exposure on Gene Expression

**Figure 3:**
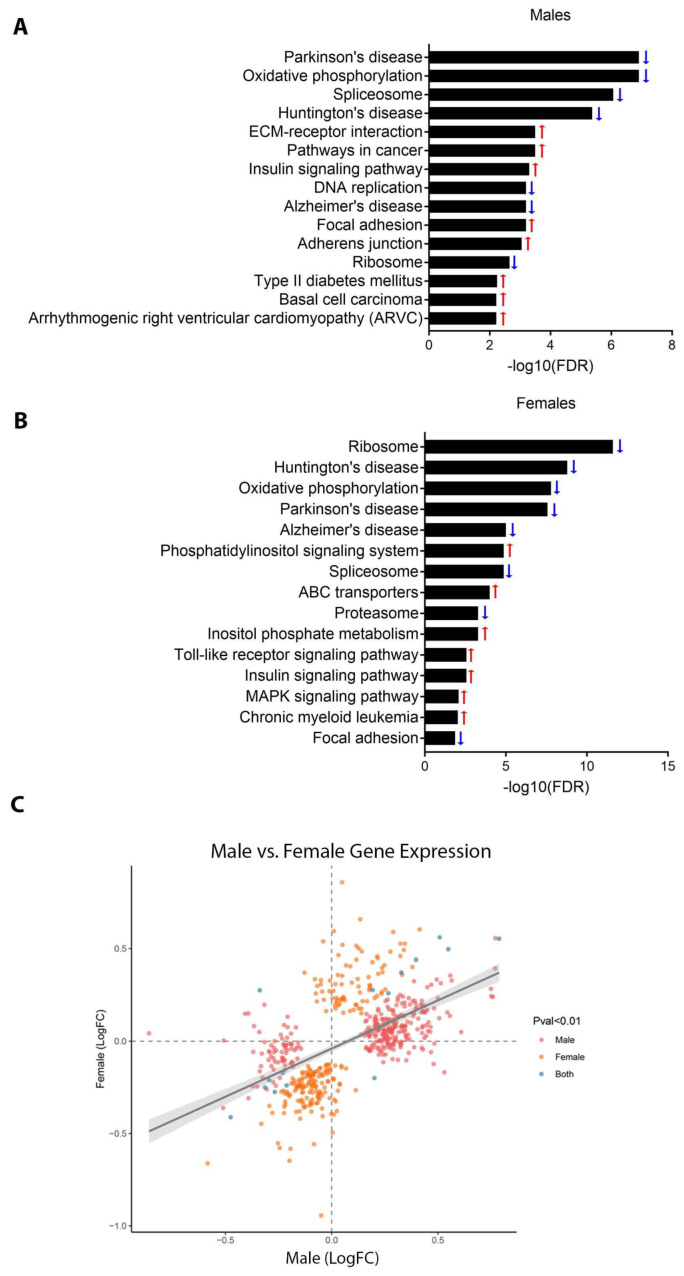
RNA-seq data in weanling mouse hearts. (**A**,**B**) Results of KEGG pathway analysis of RNA-seq data, showing the top 15 most significant pathways for males (**A**) and females (**B**). Pathways are listed in order of decreasing significance from top to bottom (i.e., smallest to largest FDR). Blue and red arrows indicate reduced and increased expression of the pathway, respectively. (**C**) Scatter plot of all genes differentially expressed (DE) with Pb exposure with a *p*-value < 0.01, depicting correlations in log2 fold change with Pb exposure between males and females. Pink, orange and blue dots depict DE genes with a *p*-value <0.01 in males, females, or both, respectively. The *p*-value for correlation between males and females was determined using Pearson’s product-moment correlation.

In order to determine the effects of perinatal Pb exposure on genome-wide transcription, we conducted RNA-seq on whole hearts isolated from male and female mice at weaning, followed by sex-stratified differential expression analysis. No individual genes met the FDR cutoff of less than 0.05 (Supplementary Tables S1 and S2). We next performed pathway analysis to determine whether biological pathways were significantly differentially expressed (see methods). The top 15 most significant KEGG pathways for each sex are shown in Figure 3A,B. Detailed KEGG pathway analysis results can be found in Supplementary Tables S3 and S4. Pathways related to metabolism, cellular energetics, and intercellular communication were significantly enriched in both sexes. Of these 15 pathways, 8 of them were significantly differentially expressed in both males and females, with the directionality of Pb-induced change being the same for both sexes. Of particular relevance to cardiac function, among these pathways were oxidative phosphorylation, insulin signaling, and focal adhesions [61,62,63] (Figure 3A,B). Several additional cardiovascular development and disease-relevant pathways were enriched only in males (adherens junction [64], type II diabetes mellitus [65], and arrhythmogenic right ventricular cardiomyopathy) or only in females (phosphatidylinositol signaling [66], ABC transporters [67], proteasome [68], and Toll-like receptor signaling [69,70]). We next determined whether gene expression changes with Pb exposure were correlated between sexes. To this end, we identified all genes with a *p*-value < 0.01 in both sexes, and plotted the log fold changes in a scatter plot (Figure 3C). Only 15 genes exhibited a *p*-value < 0.01 in both sexes, but fold changes in expression with Pb exposure for males and females were highly correlated (*p*-value < 2.2 × 10^−16^, Figure 3C). Together, these data suggest that Pb affects the expression of disease-relevant gene pathways, with effects that are both sex dependent and independent.

### 3.2. Effects of Perinatal Pb Exposure on DNA Methylation

**Figure 4:**
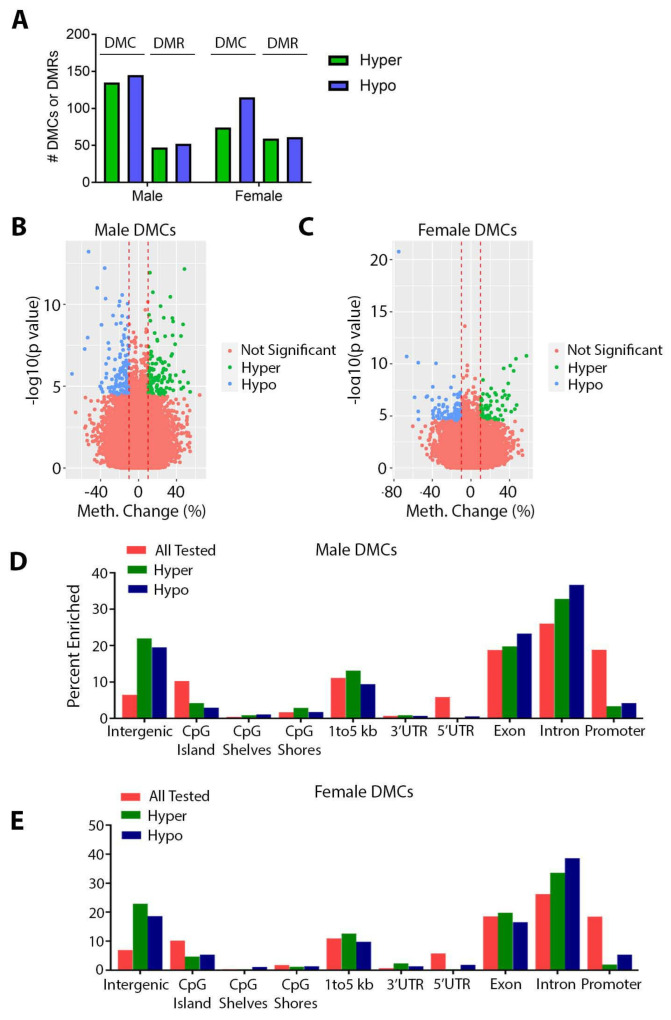
ERRBS in weanling offspring mouse hearts. (**A**) Numbers of differentially hyper- and hypomethylated cytosines (DMCs) and regions (DMRs) in male and female hearts. (**B**,**C**) Volcano plots depicting DMCs in males and females. CpGs with FDR <0.05 and at least 10% absolute change in DNA methylation were considered significant. (**D**,**E**) Annotation summary plots depicting the total number of CpGs tested in pink, hypermethylated DMCs in green, and hypomethylated DMCs in blue for each genomic annotation using the R annotatr package for males (**D**) and females (**E**).

We next examined the effects of Pb on DNA methylation in offspring hearts using ERRBS. Although no genes met the FDR criteria for significant differential expression, we observed statistically significant changes in DNA methylation at several hundred loci.In males, we identified 280 significant DMCs (Table 1, Supplementary Table S5 and Figure 4A). We additionally evaluated 1000 bp regions and identified 99 DMRs (Table 2, Supplementary Table S6 and Figure 4A). In females, we observed 189 DMCs and 121 DMRs (Table 1 and Table 2, Supplementary Tables S7 and S8 and Figure 4A). In both sexes, we observed slightly more hypomethylated DMCs and DMRs compared to hypermethylated (Figure 4A). The majority of significant changes in DNA methylation were between 10–40%; however, several loci exhibited changes that were much higher, with absolute changes as high as 70% and 74.9% in males and females, respectively (Figure 4B,C). Volcano plots of DMRs are shown in Supplementary Figure S1A,B and show similar results (absolute changes as high as 66.5% and 80.5% in males and females, respectively). We then annotated all of the CpGs covered in ERRBS for each sex to the mouse mm10 genome. For both sexes, the largest proportion of CpGs fell within exons, introns, promoters, intergenic regions, and regions 1–5 kb upstream of the transcription start site (Figure 4D,E). Compared to all of the CpGs tested, DMCs were found at a lower percentage in promoters and CpG islands and were enriched in intergenic and intronic regions of the genome in both males and females (Figure 4D,E). As expected, this pattern was also present in DMRs (Supplementary Figure S1C,D).

### 3.3. Sex Specificity of Pb-Induced Changes in DNA Methylation

**Figure 5:**
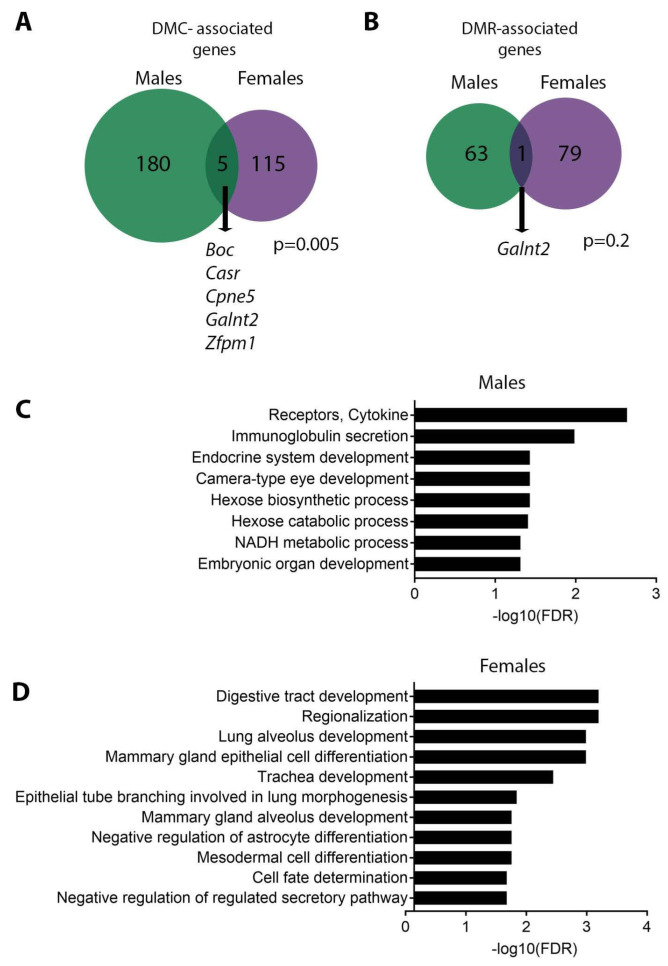
Sex specificity of Pb-induced changes in DNA methylation. (**A**,**B**) Venn diagrams depicting overlap between males and females in DMC-associated genes (**A**) or DMR-associated genes (**B**). *p*-values for overlap between the two groups calculated using a hypergeometric test. Overlap between sexes in DMC-associated genes was significant. (**C**,**D**) Polyenrich pathway analysis for DMCs in males (**C**) and females (**D**).

Changes in DNA methylation with Pb exposure at weaning were, as in adulthood [44], highly sex-specific, with no DMCs or DMRs directly overlapping between sexes. In males, 185 and 64 DMCs and DMRs, respectively, mapped to genes, while in females 120 and 80 DMCs and DMRs, respectively were associated with genes (Figure 5A,B). When comparing the genes associated with the DMCs, 5 genes were found to be in common between sexes (Figure 5A and Table 3). The overlap in DMC-associated genes, albeit small, was statistically significant (*p* = 0.005, hypergeometric test). For DMRs, males and females had a single gene, *Galnt2*, in common, which was also present in the analysis of DMCs (Figure 5B and Table 4). This overlap was not statistically significant (*p* = 0.20, hypergeometric test). Among the genes in common between sexes, *Cpne5* and *Galnt2* showed changes in DNA methylation that were in the same direction in both sexes (Table 3 and Table 4). We then examined whether Pb altered DNA methylation at previously published sex-biased genes conserved between mouse and human hearts (Supplementary Table S9 and Ref. [71]). In males, Pb exposure resulted in altered DNA methylation at only 1 male-biased gene, *Bsn*, and in females, *Trim9* was the only female-biased gene differentially methylated with Pb exposure (Supplementary Table S10). Thus, although changes in DNA methylation with Pb exposure were sex-specific, the changes did not occur at reported sex-biased genes. Given the sex specificity of Pb-induced differential DNA methylation, we hypothesized that the molecular pathways enriched among DMCs would differ by sex. To this end, we conducted sex-stratified pathway analysis using Polyenrich. After removing redundant GO terms, this analysis revealed enrichment for multiple developmental processes in both males and females, in addition to cytokine receptors, hexose biosynthesis, and NADPH metabolism in males only (Figure 5C,D). Full results of the pathway analysis are shown in Supplementary Tables S11 and S12. Overall, these results demonstrate that Pb-induced changes in DNA methylation differed substantially based on sex.

### 3.4. DNA Methylation at Weaning vs. 5 Months of Age

**Figure 6:**
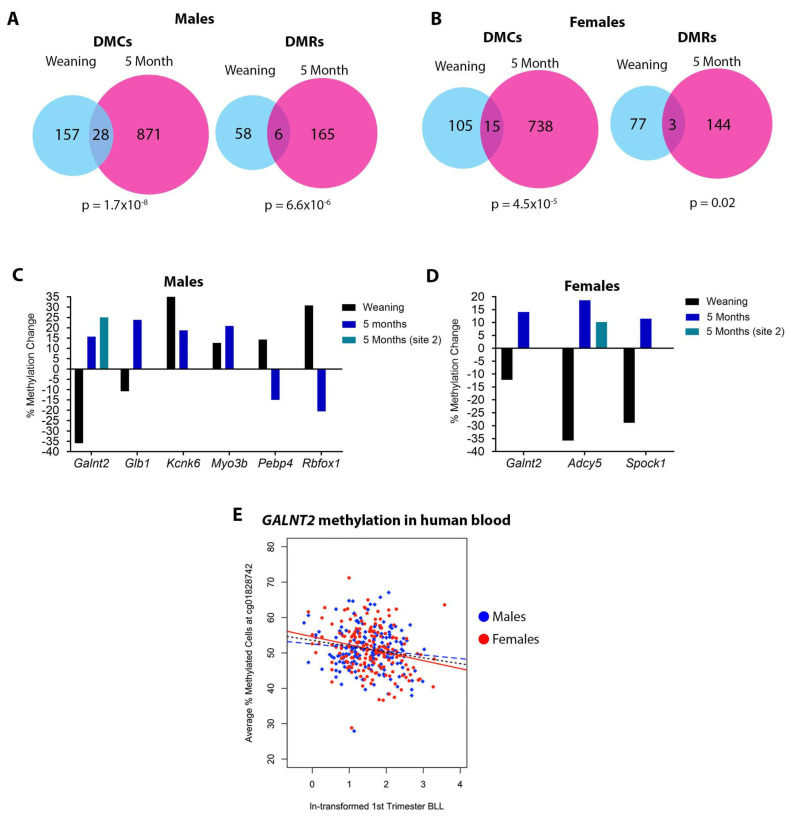
Comparison of Pb-induced DNA methylation changes between weaning and 5 months of age. (**A**,**B**) Venn diagrams depicting overlap in DMC- and DMR-associated genes between cohorts sacrificed at weaning and 5 months of age in males (**A**) and females (**B**). (**C**,**D**) bar plots depicting the direction of methylation change in DMRs, comparing the weaning and 5-month cohorts. *Galnt2* and *Adcy5* had 2 DMRs at 5 months of age. (**E**) First-trimester blood Pb levels (BLL) vs. *GALNT2* methylation in adolescent blood leukocyte DNA. One CpG site annotated to an intron of *GALNT2* was statistically significantly associated with Pb when adjusting for gender, batch, and blood cell composition (*p* = 0.00009). Red points and line: Female subjects and regression line. Blue points and line: Male subjects and regression line. Black line: Regression line for both sexes combined.

We previously reported that developmental Pb exposure results in sex-specific alterations in cardiac DNA methylation at 5 months of age [44]. Given the relative heritability and stability of DNA methylation [72], we hypothesized that many changes we observed in the weanling cohort of mice would also be present in the cohort of mice sacrificed at 5 months of age. We first looked at whether overlap existed between the two time points in DNA methylation at specific chromosomal locations, and found no directly overlapping sites in either males or females. We next looked at the genes mapping to the differentially methylated CpGs or regions. In males, 28 DMC-associated genes and 6 DMR-associated genes exhibited differential methylation in both the weanling and 5-month cohorts (Supplementary Tables S13 and S14 and Figure 6A). In females, 15 DMC-associated genes and 3 DMR-associated genes were differentially methylated in both cohorts (Supplementary Tables S15 and S16 and Figure 6B). Overlaps in DMCs and DMRs were statistically significant for both males and females (Figure 6A,B). We took a closer look at the overlapping DMR-associated genes and found that although there was differential DNA methylation at both time points, only two genes, *Kcnk6* and *Myo3b,* both identified in males only, showed changes in DNA methylation in the same direction at both time points (Figure 6C,D). All of the DMR-associated genes found to be differentially methylated at both time points play important roles in cardiovascular development and/or disease (Table 5).

A single gene, *Galnt2*, showed differential methylation in both sexes and at both time points. Although this gene was not significantly differentially expressed at weaning, expression was significantly higher in females at 5 months of age [44]. Collectively, these results suggest that a small but statistically significant number of genes showed differential DNA methylation at weaning, immediately after cessation of Pb exposure, and later in life, long after the exposure had been discontinued.

### 3.5. Effects of Gestational Pb Exposure on GALNT2 Methylation in a Human Cohort

Given the observation of differential methylation at the *Galnt2* locus in both sexes at weaning and early adulthood in mice, we considered whether this locus may also be labile to Pb exposure in humans during adolescence, a time period when Pb exposure results in increased cardiometabolic risk factors [37,73]. To this end, we investigated associations between first-trimester Pb exposure and DNA methylation at 146 CpGs in *GALNT2* in adolescent blood leukocyte samples from the ELEMENT cohort (see methods). The cohort consisted of 179 males and 186 females with an average age of 13.8 years (SD = 1.9; range 11–18 years). Maternal blood Pb concentrations had a geometric mean of 4.58 μg/dL (GSD 1.95) with a large range (0.8 to 35.8 μg/dL). After adjusting for confounders, Pb was associated with DNA methylation of one CpG using a strict Bonferonni cut-off, with the trend of the association being the same for both males and females (Figure 6E). This CpG (probe ID cg01828742) is in the first intron of *GALNT2* and is within an expected DNAseI hypersensitivity site. There was a 1.9% decrease (SE = 0.5%) in methylation with each natural-log transformed unit increase in Pb concentration at this locus. Maternal blood Pb levels were associated with 10 CpGs at a nominal *p*-value < 0.05 (Supplementary Table S17). 

## 4. Discussion

In this work, we demonstrate that Pb exposure during pregnancy and lactation in mice leads to changes in the expression of disease-relevant gene pathways and DNA methylation in offspring hearts at weaning. This work corroborates our recent study demonstrating changes in cardiac DNA methylation in adulthood [44] and adds to an important gap in our understanding of how environmental exposures impact the cardiac epigenome. Moreover, we demonstrate significant sex differences in these effects, underscoring the importance of considering sex as a biological variable in environmental health studies. Finally, we identify the CVD-relevant *Galnt2* gene as a potential biomarker of Pb exposure across sexes in both humans and mice. 

### 4.1. Pb Effects on Body Weight

In this study, we observed that Pb-exposed females were significantly heavier at weaning compared to control animals. This is consistent with work from our lab and others showing that post-natal and perinatal Pb exposures are associated with weight gain in rodent models [74,75,76]. Human studies have also demonstrated a potential role for Pb exposure in both increased and decreased body weight [73,77,78]. In the identically treated cohort of mice sacrificed at 5 months of age [39], we observed no significant Pb-induced changes in body weight at sacrifice; however, as this TaRGET study was not longitudinal, it is difficult to draw conclusions from our data about how Pb impacts body weight across the life course within individual mice. Notably, in a previous study from our laboratory which had a longitudinal design, we demonstrated that the same Pb exposure paradigm in mice led to increased body weight in males but not females in adulthood, long after cessation of exposure [75]. Collectively, our findings add to a growing body of evidence demonstrating that Pb exposure is associated with metabolic disruptions and changes in body weight.

### 4.2. Effects of Pb on Gene Expression Pathways

Although we did not find individual genes that met the threshold for differential expression with Pb exposure, we observed significant changes in the expression of several disease-relevant pathways in both males and females. In contrast to changes in DNA methylation, affected gene pathways exhibited more concordance between sexes. In both males and females, significant changes were observed in pathways related to oxidative phosphorylation, insulin signaling, and focal adhesions. Importantly, each of these three processes has been identified as critically important for normal cardiac function [61,62,63] and also targets Pb exposure [75,79,80,81]. In males, pathways associated with adherens junction, type II diabetes mellitus, and arrhythmogenic right ventricular cardiomyopathy were significantly altered by Pb exposure, while in females, we observed differential expression of pathways related to phosphatidylinositol signaling, ABC transporters, proteasome, and Toll-like receptor signaling [57,58]. Again, each of these pathways is highly relevant to cardiac development, function, and disease [64,65,66,67,68,69,70]. Moreover, previous studies have demonstrated that Pb interferes with phosphatidylinositol signaling and Toll-like receptor function, and may play a role in type II diabetes [82,83,84]. Likewise, several ABC transporters have been implicated in conferring heavy metal resistance in plants [85,86]. Upregulation of this pathway, as we observed in female hearts, may thus represent an important mechanism for adaptation to the stress of Pb exposure in mammals. In addition to several pathways clearly linked to cardiac function and disease, in both males and females, KEGG pathway analysis revealed enrichment for genes associated with Huntington’s, Parkinson’s and Alzheimer’s diseases. Interestingly, all three neurodegenerative diseases are also associated with cardiac pathology, potentially due to degeneration of the autonomic nervous system [87,88,89]. As Pb has well-established deleterious effects on the autonomic nervous system, it is plausible that the cardiac effects of Pb are due to autonomic dysfunction in addition to direct insult to the heart.

### 4.3. Effects of Pb on DNA Methylation

We have demonstrated here that, as in adulthood [44], mice exposed to Pb during gestation and lactation exhibit sex-specific alterations in cardiac DNA methylation at weaning. To our knowledge, the effects of Pb exposure on cardiac DNA methylation have not been reported elsewhere. However, our findings are consistent with studies demonstrating sex-dependent changes in DNA methylation in other tissues, including the brain, liver, blood, skin, and placenta [34,35,39,90,91]. Pathway analysis in both sexes revealed enrichment for developmental pathways, a finding that is in keeping with the role for DNA methylation in the regulation of early development. In addition to developmental pathways, several cardiovascular disease-relevant pathways were enriched among DMCs. In males, these included additional pathways related to inflammatory/immune system function (cytokine receptors and immunoglobulin secretion) and metabolism (NADH and hexose metabolism), while in females, pathways associated with developmental patterning (regionalization) showed enrichment. Notably, in both males and females, alterations in DNA methylation occurred primarily in intronic and intergenic regions of the genome. The effects of DNA methylation changes in these regions on gene expression are unclear; however, recent work suggests that changes in intronic DNA methylation are associated with CVDs [10]. The alterations in DNA methylation observed in this study were not accompanied by significant changes in gene expression. However, given the relative stability of DNA methylation, and the evaluation of a single time point in this study, it is possible that alterations in gene expression may have occurred at an earlier stage of development. Likewise, recent work suggests that environmental exposures may confer “silent” epigenetic programming on target genes, making them hyper-responsive to secondary stressors in the absence of changes in basal expression. Further studies are necessary to determine how the observed changes in DNA methylation impact gene expression.

### 4.4. Pb Exposure and Galnt2 Methylation

Although stable, DNA methylation patterns change across time with normal aging and stochastic epigenetic drift, and the stability of this mark at specific loci may be influenced by environmental exposures [92,93]. We investigated whether any Pb-induced changes in DNA methylation at weaning were also present in a separate cohort of mice sacrificed at 5 months of age, and we hypothesized that common genes may be altered at both time points. To this end, we compared DMC and DMR-associated genes in each sex and time point and found that a small but statistically significant number of genes exhibited differential DNA methylation at both weaning and adulthood. Notably, a single gene, *Galnt2*, showed differential methylation with Pb exposure in both sexes and time points, suggesting that this gene may represent an important biomarker and/or mechanistic target of Pb exposure in the heart. GALNT2 plays important roles in lipid homeostasis [94], and differential methylation of the *GALNT2* locus in humans is associated with coronary heart disease [95], and with sex differences in cardiometabolic diseases [96]. Interestingly, Pb exposure has been associated in human populations with elevated total and LDL cholesterol [97], suggesting that Pb may be linked to CVD in part through dysregulation of lipid metabolism. Altered DNA methylation of the *GALNT2* locus has also been reported in the blood of infants exposed to maternal smoking [98], and methylation of this gene in the placenta is associated with cadmium exposure [99]. The *GALNT2* gene is also differentially expressed in the peripheral blood mononuclear cells of adult smokers [100], suggesting that this gene may represent an important biomarker of multiple environmental exposures. In the ELEMENT cohort, we observed an inverse association between first-trimester Pb exposure and DNA methylation in an intron of *GALNT2* in samples from adolescents. These data suggest that *GALNT2* methylation could be a robust biomarker of early life Pb exposure across species and tissues, stable over time, but validation in additional cohorts will be necessary. The EPIC array used in the human study does not cover all CpG sites; whether the locus detected here represents a broader DMR needs to be tested using targeted analyses of the region. Importantly, Pb concentrations in the human study (range 0.8 to 35.8 μg/dL) overlap with exposure levels (16–60 μg/dL expected blood Pb levels) from the mouse model, showcasing the relevance of our mouse model. Whether *Galnt2* may be mechanistically linked to Pb-induced cardiovascular effects has not yet been investigated, and is an important area for future investigation. Likewise, the cardiovascular health implications for the observed Pb-induced effects on gene expression and DNA methylation highlighted in this study are currently unclear. Additional experiments are underway to determine the effects of perinatal Pb exposure on cardiac function across the life course and assess whether Pb-induced functional effects are mechanistically linked to altered epigenetic programming.

### 4.5. Limitations of the Study

There are several important limitations to this study. First, in this work, we investigated DNA methylation and gene expression in bulk heart tissue. The heart is comprised of several cell types, including atrial and ventricular myocytes, fibroblasts, endothelial cells, pericytes, immune cells, and neuronal cells [101]. It is likely that Pb may have effects on the heart that are highly cell-type specific, as has been reported for other tissues [102,103]. Single-cell studies of Pb effects on the heart may yield additional important insight into the mechanisms by which Pb induces CVDs. A second limitation of this work is the use of ERRBS for the analysis of DNA methylation. Although ERRBS is far more economical than whole genome approaches, this method captures just a small fraction of CpGs [50]; therefore, it is possible that some Pb-induced changes in DNA methylation not covered by this approach may have been missed. Moreover, ERRBS relies on traditional bisulfite sequencing, which cannot distinguish between DNA methylation (5-methylcytosine) and DNA hydroxymethylation (5-hydroxymethylcytosine). DNA hydroxymethylation plays an important role in normal cardiovascular development, is dysregulated in CVDs, and is also labile to environmental exposures [10,104]. Thus, future studies into the sex-specific effects of Pb and other toxicant exposures on DNA hydroxymethylation in the heart are warranted. Finally, as this TaRGET II Consortium study was focused on the epigenomic effects of Pb exposure, we did not assess the effects of Pb exposure on cardiac function. However, studies are currently underway to understand how developmental Pb exposure affects long-term cardiovascular function, and to identify potential mechanistic links between adverse health effects and changes to the cardiac epigenome. 

**Table 5:** Literature summary of DMR-associated genes at weaning that also show differential methylation in mice at 5 months of age.

Gene	Differential Methylation in Males or Females?	Link to Cardiovascular Development or Disease	Reference
Galnt2	Both	Regulation of HDL cholesterol; promoter hypermethylation associated with coronary heart disease; DNA methylation at GALNT2 associated with sex differences in cardiometabolic diseases	[94,95,96,105]
Glb1	Males	Mutation of GLB1 associated with cardiomyopathy in humans	[106]
Kcnk6	Males	Channel encoded by this gene functions in ventricular repolarization; Gene deficiency in mice leads to pulmonary hypertension	[107,108]
Myo3b	Males	Expressed in cardiac mesoderm stage of cardiac differentiation	[109]
Pebp4	Males	Expressed in adventitial layer of coronary arteries	[110]
Rbfox1	Males	Functions in cardiac gene splicing and is down-regulated in heart failure; copy number variants associated with stress cardiomyopathy; gene variants associated with lower blood pressure	[111,112,113,114]
Adcy5	Females	Adenylyl cyclase 5 exacerbates oxidative stress and cardiomyopathy in response to chronic adrenergic stimulation; knockout of Adcy5 in mice protects against aging-induced cardiomyopathy; enhances myocardial contractility and function during exercise	[115,116,117]
Spock1	Females	Plays a role in calcification of the vasculature; and in collagen deposition during cardiac fibrosis	[118,119]

## 5. Conclusions

Although animal and human epidemiologic studies implicate Pb exposure in various CVDs, the underlying molecular mechanisms are incompletely understood. In particular, the sex-specific effects of developmental toxicant exposures on the cardiac epigenome have not been investigated. In this work, we demonstrate that Pb exposure leads to hundreds of sex-specific differentially methylated cytosines and regions in the hearts of mice at weaning, with enrichment for pathways associated with early development. We further demonstrate significant changes in gene expression at CVD-relevant pathways in both sexes. These data collectively suggest that developmental Pb exposure, through programming of the epigenome and gene expression, may render the heart more vulnerable to disease and injury across the life course, a question that will be investigated in future studies.

## Figures and Tables

**Table 1:** Numbers of differentially methylated cytosines (DMCs) in weanling mouse hearts.

Condition	Total	# Hypermethylated (% Total)	# Hypomethylated (% Total)	Total Tested
Female Pb	189	74	115	672,230
Male Pb	280	135	145	730,731

**Table 2:** Numbers of differentially methylated regions (DMRs) in weanling mouse hearts.

Condition	Total	# Hypermethylated (% Total)	# Hypomethylated (% Total)	Total Tested
Female Pb	120	59	61	137,035
Male Pb	99	47	52	134,644

**Table 3:** DMC-associated genes exhibiting differential DNA methylation in both males and females.

Gene	% Change DNA Meth. (M)	FDR (M)	Genomic Annot. (M)	Chromosomal Position (M)	% Change DNA Meth. (F)	FDR (F)	Genomic Annot. (F)	Chromosomal Position (F)
Boc	25.1	6.99 × 10^−3^	Exon	44496428	−16.96	0.010	Exon	44496335
Casr	17.14	0.014	Intron	36530044	−19.68	0.030	Intron	36530087
Cpne5	−25.34	0.042	1 to 5 kb	29241524	−58.46	2.26 × 10^−3^	1 to 5 kb	29241568
Galnt2	−15.39	0.037	Intron	122767052	−15.76	6.63 × 10^−3^	Intron	122335932
	27.41	0.011	Intron	122336116	−10.77	1.39 × 10^−3^	Intron	121128632
					11.12	0.012	Intron	121567725
					13.59	0.050	Intron	122789211
					39.47	1.89 × 10^−3^	Intron	122698947
Zfpm1	27.41	0.011	Exon	122336116	−15.76	6.63 × 10^−3^	Exon	122335932

**Table 4:** DMR-associated gene exhibiting differential DNA methylation in both males and females.

Gene	% Change DNA Methylation (M)	FDR (M)	Genomic Annotation (M)	Chromosomal Start Position (M)	% Change DNA Methylation (F)	FDR (F)	Genomic Annotation (F)	Chromosomal Position (F)
Galnt2	−35.94	0.001	Intron	120754001	−12.23	0.038	Intron	123893001

## Data Availability

ERRBS and RNA-seq data will be uploaded to GEO. Additional data that support the findings of this study are available from the corresponding author, Laurie K. Svoboda, upon reasonable request. The human datasets supporting the conclusions of this article are not publicly available due to human subjects’ protections. The de-identified data are available upon reasonable request to author Karen E. Peterson (karenep@umich.edu) following review and approval by the ELEMENT Executive Committee.

## Supplementary Materials

The following supporting information can be downloaded at: https://www.mdpi.com/article/10.3390/toxics11010085/s1, Table S1: RNA-seq analysis in Pb-treated males; Table S2: RNA-seq analysis in Pb-treated females; Table S3: Full results of KEGG pathway analysis in males; Table S4: Full results of KEGG pathway analysis in females; Table S5: DMCs in Pb-treated males; Table S6: DMRs in Pb-treated males; Table S7: DMCs in Pb-treated females; Table S8: DMRs in Pb-treated females; Table S9: Sex biased genes shared between mouse heart and human left ventricle - From Deegan et al., Biol of Sex Diff, Additional File 15; Table S10: Changes in DNA methylation at sex-biased genes (from Deegan et al., 2019); Table S11: Polyenrich pathway analysis of male DMCs; Table S12: Polyenrich pathway analysis of female DMCs; Table S13: Male DMC-associated genes showing differential methylation at both weaning and 5 months of age; Table S13: Male DMR-associated genes showing differential methylation at both weaning and 5 months of age; Table S15: Female DMC-associated genes showing differential methylation at both weaning and 5 months of age; Table S16: Female DMR-associated genes showing differential methylation at both weaning and 5 months of age; Table S17: Associations between first trimester Pb and blood leukocyte DNA methylation at CpG Sites in GALNT2, from the ELEMENT cohort; Figure S1: Plots of differentially methylated regions (DMRs) in offspring mouse hearts.
