# Supplementary material for: Perinatal Lead Exposure Promotes Sex-Specific Epigenetic Programming of Disease-Relevant Pathways in Mouse Heart
Source: Toxics. 2023 Jan 16;11(1):85. doi: 10.3390/toxics11010085 (PMC9860846; doi:10.3390/toxics11010085)
Supplement: Supplementary file 1 [file toxics-11-00085-s001.zip › toxics-2078294-supplementary.pdf]

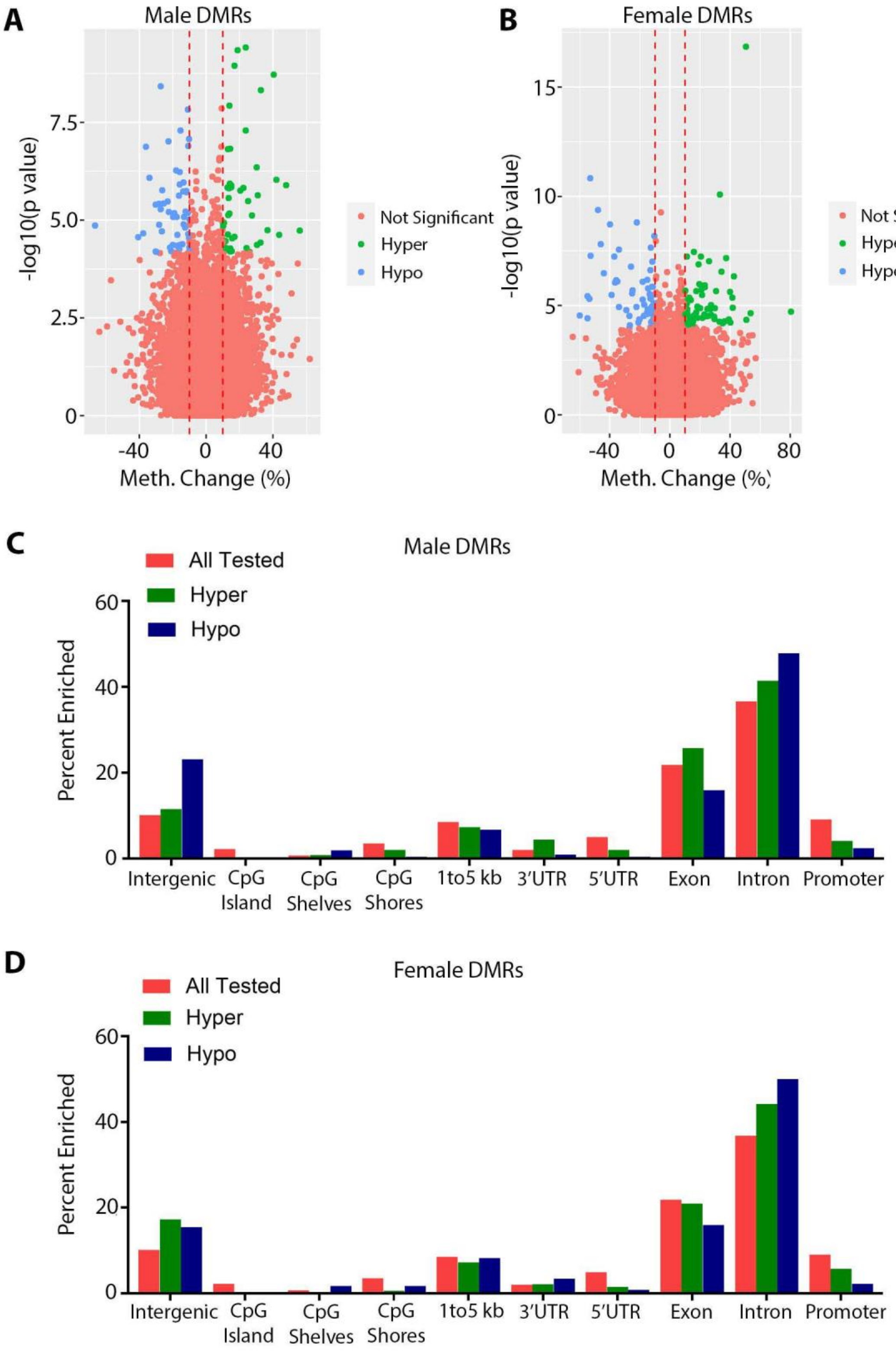

Figure S1. Plots of differentially methylated regions (DMRs) in offspring mouse hearts. (A-B) Volcano plots depicting DMRs (1000 base pair segments of the genome) in males and females. Regions with FDR <0.05 and at least 10% absolute change in DNA methylation were considered significant. (C-D) Annotation summary plots depicting the total number of CpGs tested in pink, hypermethylated DMRs in green, and hypomethylated DMRs in blue for each genomic annotation using the R annotatr package for males (C) and females (D).
